# For an ethnomedicine enriched by human diversity

**DOI:** 10.31744/einstein_journal/2022CE6083

**Published:** 2022-02-23

**Authors:** Olukemi Adeola Ribeiro Salami

**Affiliations:** 1 Universidade Paulista São Paulo SP Brazil Universidade Paulista, São Paulo, SP, Brazil.

Dear Editor,

I began my school life in a white institution thanks to the good socio-economic status achieved by my parents after years of hard working. At the school, I was always the only black girl in the classroom. For this reason, at the age of 6-years, I became aware of the perverse racism presented in our society.

However, in my multiethnic and multiracial family environment I was gifted with loving relationships and mutual commitment. For this reason, I developed mechanisms to sustains my ideal of healthy interethnic and interracial relationships.

My dual ethnical background (white Christian and black Yoruba) and my dual nationality (Brazil-Nigeria) determined my personal, educational and professional paths. In addition, these were responsible for my entering in medical school at the age of 18, without ever questioning whether that was my place. As a black woman, I occupied and continue to occupy this space under questioning eyes related to my deservingness to be there, intellectual capacity, and under frequent microaggressions from colleagues, professors, and users of the health systems.

In the history of Brazil, serious misconceptions, which reduce a human condition to legal condition, were provoked by the synonym between “black” and “slave”. This misunderstanding was fed by researchers who rely on sources produced by foreigners incapable of discriminating between the conditions of enslaved and freed blacks.^(1)^ Since the 1980s, new approaches in the historiography of slavery have made possible more accurate analyses of the Brazilian slave process, which was not enough, however, to overcome the reductionism of the history of blacks to the history of slavery.

The notion of race functions from two basic and complementary registers: the biological and the ethno-cultural.^(2)^ The latter, associated with tradictions, religion and geographical origin that make up the so-called “cultural racism”.^(3)^The action of racism is perverse and shows how negatively stereotyped representations of Africans, African descendants, and their religions are related.^(4)^

The psychosocial effects of racism shape behaviors, thoughts and feelings, on the basis of which discriminators and discriminated are differentiated. The victims, transformed into defendants, and solely responsibles for the suffering imposed on them, are conditioned to feel inferior or subhuman. The damage to psychological health includes anguish, panic, anxiety, insecurity, guilt, self-censorship, rigidity, loneliness, chemical dependencies, and syndromes. The promotion of equity, inclusion and recovery include factors related to socioeconomic and educational indices, and processes of reconstruction of subjectivities.^(5)^

In Brazil, in the health field, the National Policy for Integral Health of the Black Population aims at fighting ethnic-racial discrimination in the Brazilian Public Health System (*Sistema Único de Saúde* -SUS), defends equity in health for the black population, and has as the main guideline the “inclusion of the topics related to Racism and Health of the Black Population in the processes of training and permanent education of health workers and in the exercise of social control in health area.^(6)^

Today, graduated for almost 10 years, I witness the intensification and spread of the Black Lives Matter Movement, which has expanded around the world, and also reached the Brazilian academic elite as a wave of collective awareness about racism. I had the opportunity to talk to medical students about this subject, which, although so necessary in our historical and social context, is still faces serious difficulties of consolidation in the curricula of training courses for health professionals, and seldom appear in the medical school curriculum.^(7)^ Even more concerning are the invisibility of racism as a social determinant of health, the opportunity that is lost to prepare medical professionals for a humanized care that takes into consideration the biopsychosociocultural contexts in which patients are inserted, and the process on how these contexts affect the processes of health, disease, treatment, cure or death.

Clearly, the issue of racism will not be completely solved through medical education adequate to the Brazilian reality, but for the first time, I noticed a promising concern of young black and white future physicians engaged with racial/ethnic inequities in health area. I witness, for the first time in medicine, the impulse for the creation of strategies capable of reducing or even annullate ethno-racial inequalities in this country.

Currently, more than ever, the time has come for higher education health institutions to act in a concrete and precise manner in the implementation of disciplines, courses, and undergraduate, and graduate programs of sufficient quality to deal effectively with the theme of Afro-descendant citizenship.

To achieve this goal, it is necessary to create, in the health field, a space for qualified debates on the African presence in the multicultural and multiracial composition of the Brazilian society. Some recommendations by Monteiro^(8)^ are useful for the definition of strategies and tactics to achieve the desired goal.

Our first task is to identify the health needs and potentialities of the black population and, to accomplish this, it is necessary to articulate knowledge and methods from several areas of knowledge. For pedagogical purposes, Monteiro^(8)^ establishes five thematic axes to be considered in the continuing education of health professionals: racism, African and Afro-Brazilian history and culture, health of the black population, the body, and affirmative action practices. The articulation of themes and sub-themes around these axes results in a complex network of knowledge. Many of these knowledge come from ethnomedicine, an indispensable field for the dialogue between medicine and anthropology, between health and spirituality/religion, and between contemporary Western medicine and Afro-diasporic health care practices.

Totally aware of the history of humiliations experienced by black people, and due to my special condition as a black physician and of the commitments and responsibilities associated with my profession, I am trying to put my strength and my will at the service of justice and peace in human relations, and to the service of the health and well-being of the people with whom I share steps on my journey.
